# The influence of inlet velocity profile on predicted flow in type B aortic dissection

**DOI:** 10.1007/s10237-020-01395-4

**Published:** 2020-10-17

**Authors:** Chlöe Harriet Armour, Baolei Guo, Selene Pirola, Simone Saitta, Yifan Liu, Zhihui Dong, Xiao Yun Xu

**Affiliations:** 1 Department of Chemical Engineering, Imperial College London, London, SW7 2AZ UK; 2 Department of Vascular Surgery, Zhongshan Hospital, Institute of Vascular Surgery, Fudan University, Shanghai, China

**Keywords:** Type B aortic dissection, Computation fluid dynamics, Inlet boundary condition, Patient-specific simulation

## Abstract

In order for computational fluid dynamics to provide quantitative parameters to aid in the clinical assessment of type B aortic dissection, the results must accurately mimic the hemodynamic environment within the aorta. The choice of inlet velocity profile (IVP) therefore is crucial; however, idealised profiles are often adopted, and the effect of IVP on hemodynamics in a dissected aorta is unclear. This study examined two scenarios with respect to the influence of IVP—using (a) patient-specific data in the form of a three-directional (3D), through-plane (TP) or flat IVP; and (b) non-patient-specific flow waveform. The results obtained from nine simulations using patient-specific data showed that all forms of IVP were able to reproduce global flow patterns as observed with 4D flow magnetic resonance imaging. Differences in maximum velocity and time-averaged wall shear stress near the primary entry tear were up to 3% and 6%, respectively, while pressure differences across the true and false lumen differed by up to 6%. More notable variations were found in regions of low wall shear stress when the primary entry tear was close to the left subclavian artery. The results obtained with non-patient-specific waveforms were markedly different. Throughout the aorta, a 25% reduction in stroke volume resulted in up to 28% and 35% reduction in velocity and wall shear stress, respectively, while the shape of flow waveform had a profound influence on the predicted pressure. The results of this study suggest that 3D, TP and flat IVPs all yield reasonably similar velocity and time-averaged wall shear stress results, but TP IVPs should be used where possible for better prediction of pressure. In the absence of patient-specific velocity data, effort should be made to acquire patient’s stroke volume and adjust the applied IVP accordingly.

**Electronic supplementary material:**

The online version of this article (10.1007/s10237-020-01395-4) contains supplementary material, which is available to authorized users.

## Introduction

The choice of inlet boundary condition is crucial to ensure accuracy and validity of numerical solutions in any computational fluid dynamic (CFD) simulation. For CFD analysis of the aorta, it is important to employ a physiological boundary condition that faithfully mimics the ejection of blood from the heart through the aortic valve. CFD models of type B aortic dissection (TBAD) have often assumed idealised inlet velocity profiles (IVPs) (Alimohammadi et al. 2013; Chen et al. 2013; Cheng et al. 2010; Dillon-Murphy et al. 2015; Tse et al. 2010). With the advancement and increasing availability of imaging techniques, it has become possible to extract detailed flow and velocity profiles from patient images, including through-plane (TP) and three-directional (3D) IVPs from cine phase-contrast or 4D flow magnetic resonance imaging (MRI).

Several studies have reported the influence of different types of IVPs on flow in various regions of the cardiovascular system, including the carotid bifurcation (Campbell et al. 2012; Moyel et al. 2006; Wake et al. 2009) and coronary arteries (Myers et al. 2001). The impact of inlet boundary condition on aortic hemodynamics has also been assessed by various researchers (Chandra et al., 2013, Morbiducci et al. 2013, Pirola et al. 2018 and Youssefi et al. 2018). These studies show that the type of IVP, in the form of a spatially varying TP velocity profile or 3D profile containing all three velocity components, has a strong impact on the hemodynamics and related parameters in the ascending aorta and aortic arch, but it has limited influence on flow in the descending aorta.

All of the aforementioned aorta-based studies were conducted in non-dissected aortas. Under normal conditions, flow in the descending aorta is likely to be fairly organised. In dissection cases, the aortic geometry is very complex, with multiple channels which can extend from the primary entry tear (PET) all the way to below the aortic bifurcation. In many type B aortic dissections, the PET is located just distal to the left subclavian artery (LSA) on the aortic arch where the influence of inlet velocity profile could still be significant.

It is usually difficult to access complete sets of patient data of high enough quality to extract both the inlet velocity profile and geometry. This may be due to a lack of advanced imaging facilities in hospitals, or due to missed opportunities for certain scans because of the patient’s condition or urgency in administering treatment. It is often the case that only a CT scan acquired for diagnosis purposes is available, from which the geometry can be reconstructed for patient-specific flow simulation. In this scenario, a generic inlet flow waveform is usually applied, which does not contain patient-specific flow features, such as heart rate and stroke volume. Stroke volume is the total volume of blood ejected by the heart with each beat, which has a typical value of 94 ± 15 mL (Maceira et al. 2006). Furthermore, when estimating stroke volume from 4D MRI data, its value could vary depending on the plane on which the velocity data are extracted. Currently, there is a lack of systematic evaluation of the effect of such non-patient-specific inlet boundary conditions on predicted hemodynamic indices in aortic dissection.

This study aims to quantify the influence of various IVPs on CFD simulations of type B aortic dissection under two scenarios. The first is where patient-specific flow data is available—in this case results obtained with 3D, TP and flat IVPs are compared. The second scenario is where no patient-specific flow data is available—in this case generic flat velocity profiles are applied and the effect of non-patient-specific stroke volume and waveform is assessed.

## Methodology

**Fig. 1:**
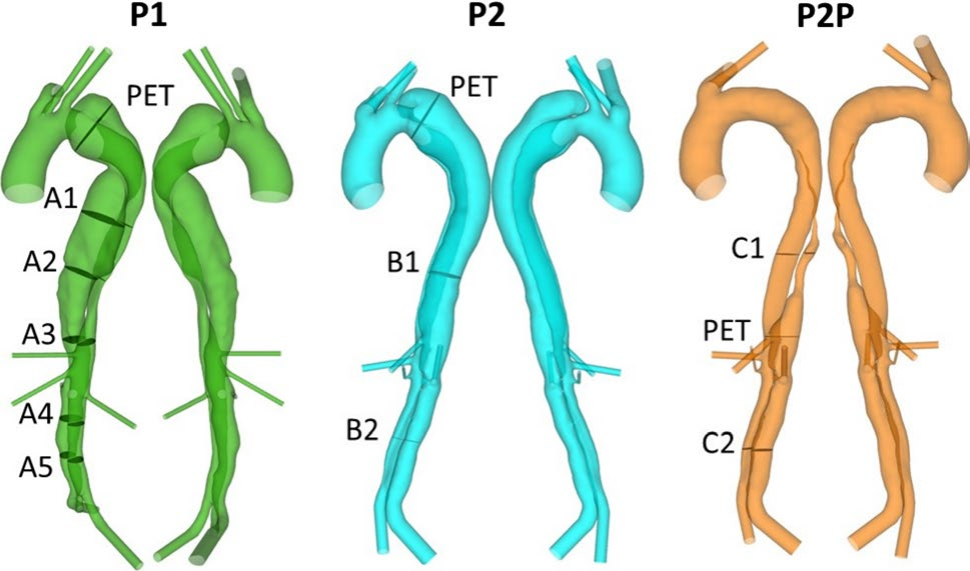
Geometries of P1, P2 and P2P used for simulation. The primary entry tear (PET) in each model is indicated, as well as planes on which pressure readings were taken (A1-5, B1-2 and C1-2)

Three sets of images acquired from two patients treated for acute type B aortic dissection (TBAD) at the Zhongshan Hospital in Shanghai, China, were used in this study. As shown in Fig. 1, P1 and P2 represent pre-TEVAR models, extracted from diagnosis CT scans, both of which have dissections extending from the level of LSA down to the aortic bifurcation. P2P is a post-TEVAR model of P2, used to represent the type of TBAD geometry with a PET further down the descending aorta. All three geometries were segmented from CT scans in Mimics (Materialise HQ, Leuven), using a range of automatic thresholding and manual segmentation methods. On the diagnosis scan of P2, partial thrombosis of the proximal false lumen was observed. Therefore, to evaluate the effect of inlet velocity profile on thrombus formation in addition to hemodynamics the initial dissection geometry was recovered by including the thrombosed section in the false lumen.

Computational meshes were created using ICEM CFD (Ansys Inc, v15.0). All meshes were unstructured and consisted of a hexahedral core, with ten prismatic layers, reducing in size towards the wall, to ensure adequate near wall resolution. Local areas of refined mesh were created in each model around tears, sharp bends leading to aortic branches, and any region of complex geometry. Mesh sensitivity tests were conducted to ensure the solution was mesh-independent. For these tests, transient flow simulations with flat IVPs were performed. Global time-averaged wall shear stress (TAWSS) patterns were first compared visually to check qualitative consistency. Mean and maximum velocity and TAWSS were then quantitatively compared at selected planes throughout the aorta, focusing on areas within the dissection and near tears. The mesh was refined until differences in these parameters between the chosen mesh and a finer mesh were less than 3.5%. The grid convergence index (CGI) was also calculated, and the chosen mesh had a CGI of < 5.5% for velocities and TAWSS at all selected planes, in line with previous studies (Craven et al. 2009; Tedaldi et al. 2018). Further details on the mesh sensitivity study can be found in the supplementary material. The final meshes contained 6.2, 5.8 and 4.1 million elements for P1, P2 and P2P, respectively.

**Fig. 2:**
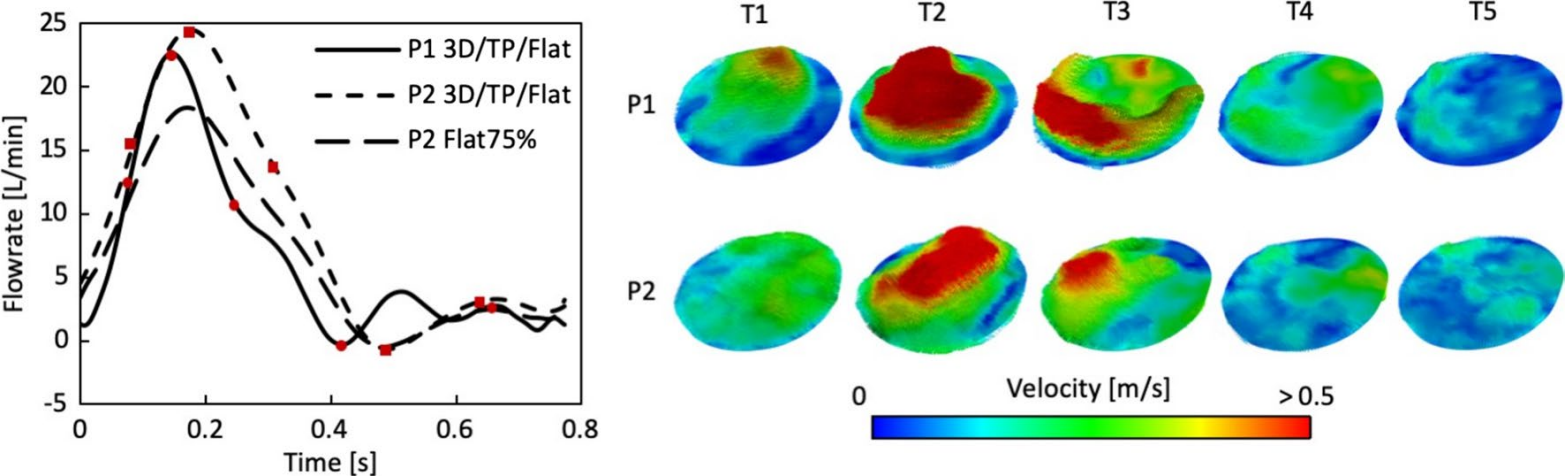
Left: Flow rate waveforms derived from 4D flow MRI of P1 and P2. Right: 3D inlet velocity profiles for P1 and P2 at time points (T1–T5) throughout the cardiac cycle. Time points are indicated by red circles and squares for P1 and P2, respectively, on flow rate curves

3D IVPs were extracted from the 4D MRI data of P1 and P2 using an in-house MATLAB processing tool, developed in our previous studies (Pirola et al. 2018, 2019). From the results obtained with the 3D IVP simulation, TP and flat velocity profiles were derived using Ansys EnSight (v10.2) and an additional in-house MATLAB tool. As post-TEVAR 4D MRI was not available for P2P, the 3D, TP and flat IVPs extracted for P2 were used. Furthermore, to assess the effect of non-patient-specific stroke volume and flow waveform, two additional IVPs were tested on P2—the flat profile for P2 with a 25% reduction in flow (Flat75%), and the flat profile for P1 (FlatP1). Flat75% and FlatP1 had a stroke volume of 87 and 85 mL, respectively, compared to the patient-specific IVPs for P2 which had a stroke volume of 115 mL. Figure 2 shows the flow waveforms for all inlet boundary conditions tested as well as the 3D IVPs for P1 and P2.

In all simulations, three-element Windkessel (3EWK) models were applied at the outlets. For P1, the parameters were tuned following the methodology of Pirola et al. (2019), which calibrates the 3EWK models based on invasive Doppler wire (DW) pressure readings and branch flows calculated from the 4D MRI data. For P2 and P2P, the same methodology for calibrating the parameters was followed based on several assumptions. First, invasive pressure measurements on P1 were adopted as these were not available for P2 or P2P. Furthermore, the 4D MRI data for P2 was only of high enough quality to extract flow rates to the arch branches. Therefore, the proportion of flow to the abdominal branches was assumed to be the same as that for P1. The 3EWK parameters were adjusted for the Flat75% and FlatP1 IVPs simulated in P2 to account for the lower inlet flow rate. As no 4D MRI scan for P2P was available, the branch flow split for P2 was assumed. Additionally, it was assumed that flow through the left common carotid artery (LCCA) in P2P was the sum of the flow through the LCCA and LSA in P2 as the LSA was occluded during the TEVAR procedure and revascularisation was not performed.

All simulations were carried out in Ansys CFX (v15.0). The blood was assumed to be a Newtonian fluid with a viscosity of 0.004 Pa s and a density of 1060 kg m^3^. The flow was assumed to be laminar based on calculations of the peak Reynolds number, Womersley number and the critical Reynolds number for transition to turbulence reported by Kousera et al. (2013). A time step of 0.001 s was selected, and all simulations were run for a minimum of four cardiac cycles to ensure periodic solutions. The final cycle was used for analysis, and post-processing and visualisation of the results were carried out in EnSight.

## Results

### Flow patterns

**Fig. 3:**
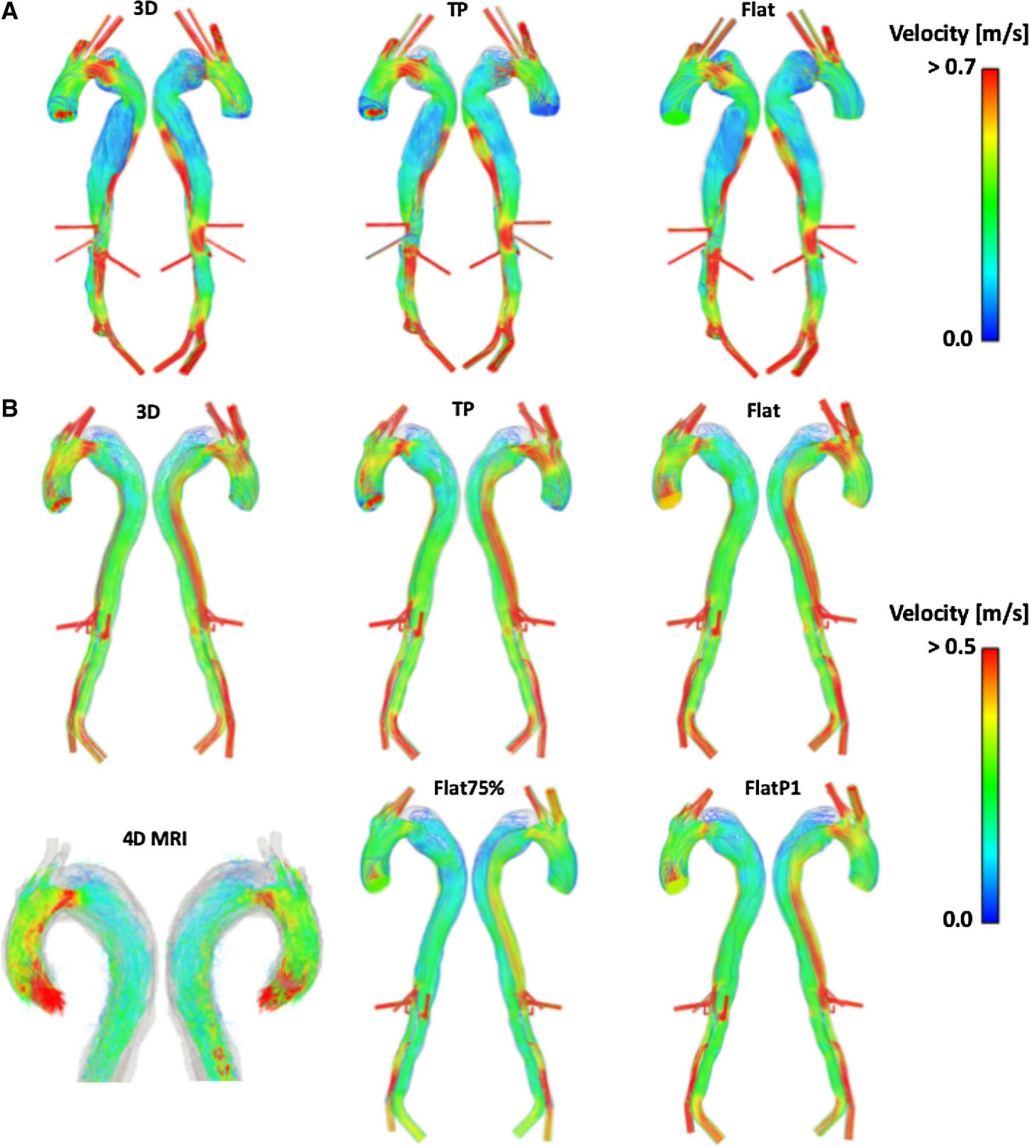
Peak systolic streamlines for **a** P1 with a 3D, TP and Flat inlet velocity profile (IVP), **b** P2 with a 3D, TP, Flat, Flat75% and FlatP1 IVP, with 4D MRI derived streamlines for comparison

**Fig. 4:**
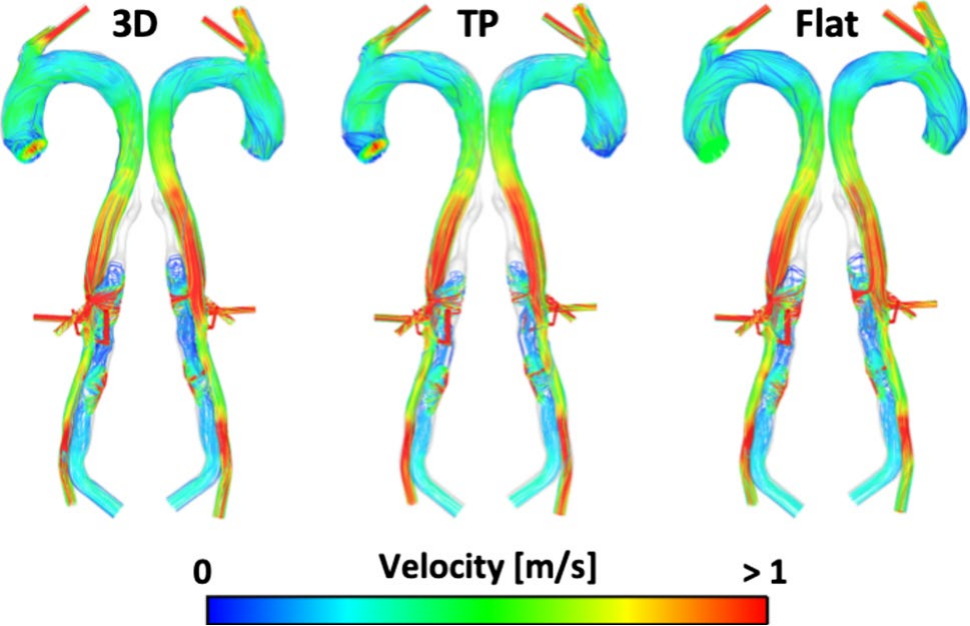
Peak systolic streamlines for P2P with a 3D, TP and Flat inlet velocity profile

Peak systolic velocity streamlines for each IVP studied can be seen in Fig. 3 for P1 and P2, and Fig. 4 for P2P. Additionally, Fig. 3 also includes the peak systolic velocity streamlines derived from the 4D MRI scan for P2. The equivalent 4D MRI data for P1 was previously reported by Pirola et al. (2019). For validation of the computational methods used throughout this study, the streamlines obtained with 3D IVP for P1 and P2 were compared to their respective 4D MRI streamlines. For both P1 and P2, the velocity streamlines show good agreement between the 3D IVP and 4D MRI. High velocity jets through the PET are captured for both patients, with the peak velocity of the 3D IVP and 4D MRI being 0.9 and 1.1 m/s (Pirola et al. 2019) for P1, respectively, and 0.6 and 0.7 m/s for P2, respectively. The velocity patterns are also well captured in the descending aorta, with lower FL velocities in P1 seen in both the 3D IVP and 4D MRI results, while the higher TL velocities observed in the 4D MRI streamlines for P2 are correctly modelled with the 3D IVP. Based on this validation, all other IVPs are compared to their respective 3D IVP results.

In all models, the streamlines do not vary drastically between the 3D, TP and flat IVPs. However, looking in detail at certain areas differences can be observed. In the ascending aortas, higher velocity values as well as more helical flow are observed with the 3D and TP IVPs compared to the flat IVP. Focusing on the PET on the aortic arch of P1 and P2, both patients see a reduced volume of high velocity through the suppressed TL with the flat IVP compared to the 3D IVP. Both patients also have an area of low velocity in the upper FL at the aortic arch. In this region, there are varied velocity patterns; however, the low flow means visual comparison is difficult as there are fewer streamlines. The difference in flow patterns is clearer in the resulting TAWSS contours in this region, which are discussed in the following section. The maximum velocity varies by < 3.5% between the 3D, TP and flat IVPs for both patients. The mean velocity in the PET varies by < 2% for P2 between the three IVPs and for P1 between the 3D and TP IVP. However, using the flat IVP in P1 results in an increase in mean velocity by 8.6%. With the Flat75% and FlatP1 inlet profiles for P2, the maximum velocity at the PET is reduced by 28% and 12%, respectively, compared to the 3D IVP. For P2P, the peak velocity through the PET differs by < 1%.

### Wall shear stress

**Fig. 5:**
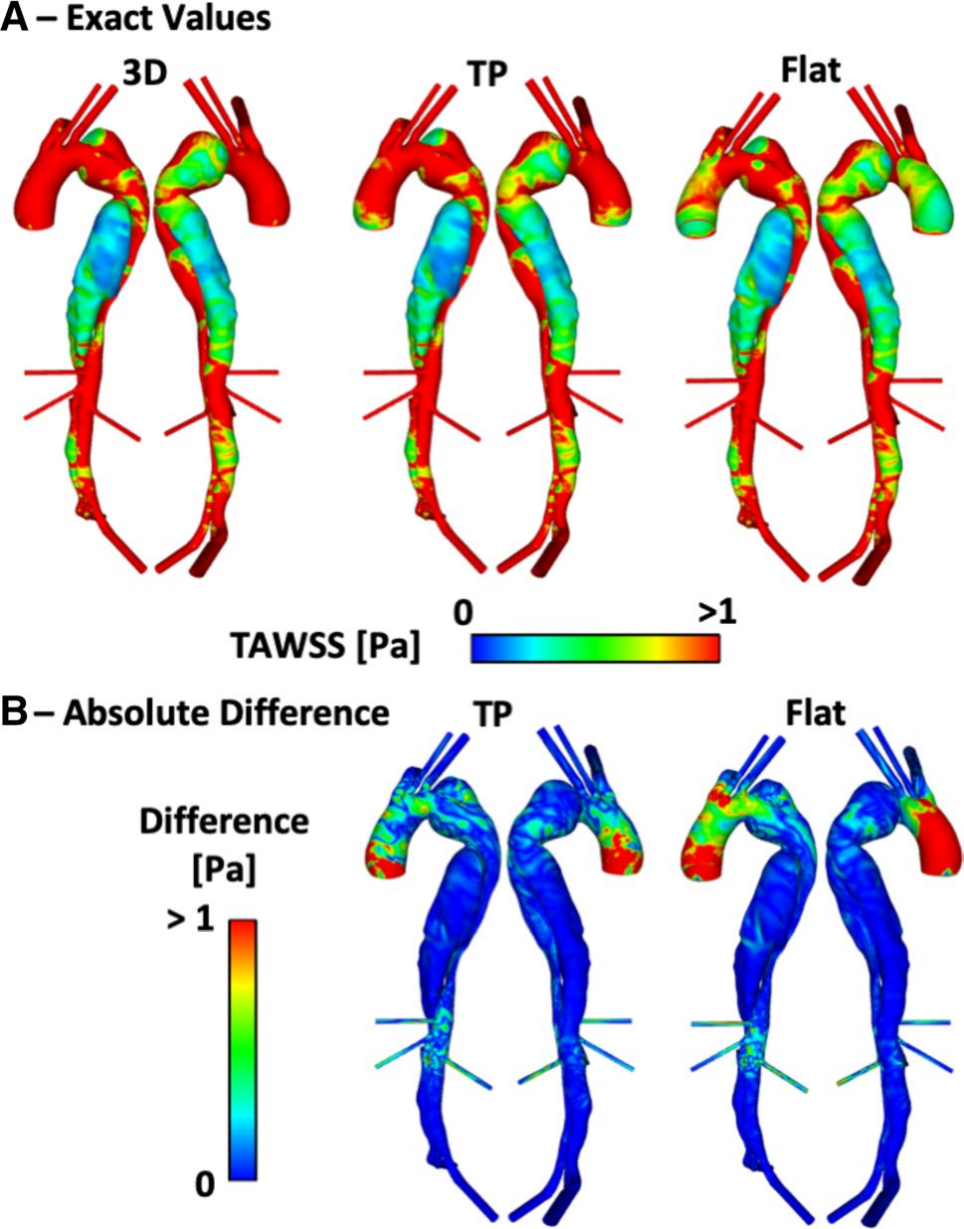
**a** P1 TAWSS values with a 3D, TP and Flat inlet velocity profile (IVP). **b** Absolute difference in TAWSS values between the 3D IVP and the two other IVPs

**Fig. 6:**
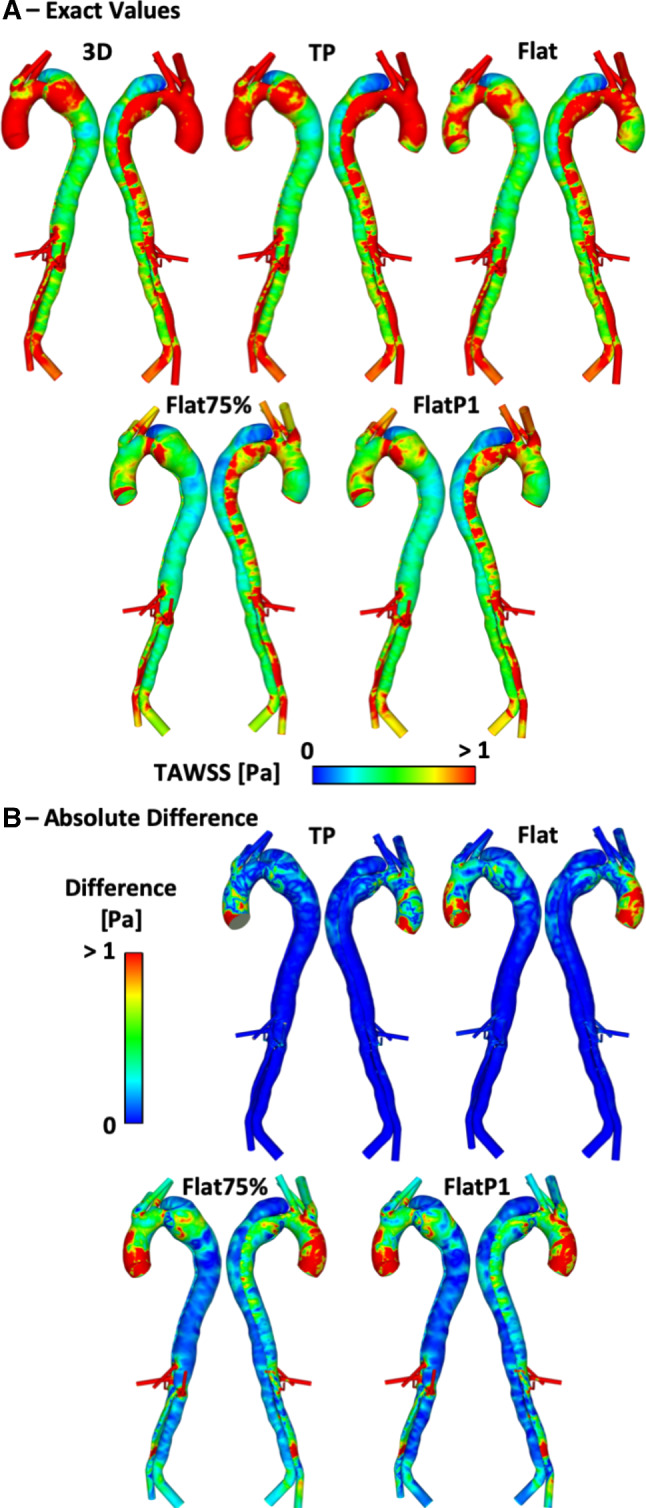
**a** P2 TAWSS values with a 3D, TP, Flat, Flat75% and FlatP1 inlet velocity profile (IVP). **b** Absolute difference in TAWSS values between the 3D IVP and the four other IVPs

**Fig. 7:**
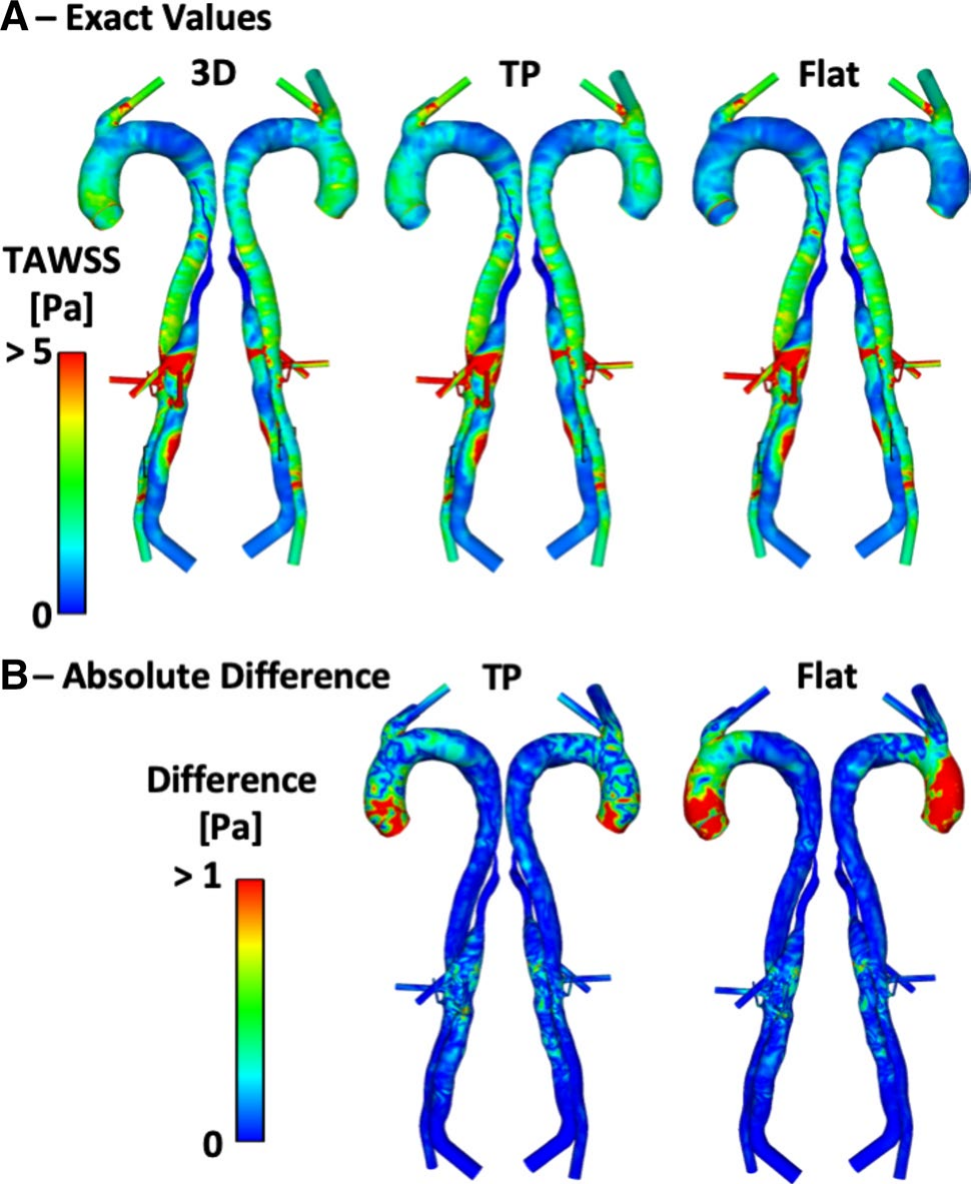
**a** P2P TAWSS values with a 3D, TP and Flat inlet velocity profile (IVP). **b** Absolute difference in TAWSS values between the 3D IVP and the two other IVPs

Figures 5, 6 and 7 show the TAWSS distributions for P1, P2 and P2P, respectively. Also shown is the absolute difference in TAWSS between each inlet profile and the gold standard 3D IVP results. As expected, large variations in TAWSS are seen in the ascending aorta and aortic arch. Throughout most of the descending aorta, there is little difference in TAWSS except in regions near the tears. Comparing the patient-specific 3D, TP and flat IVPs, near the PET on the aortic arch of P1 and P2, there are variations for TAWSS below 1 Pa, and particularly < 0.2 Pa (a key threshold value when predicting thrombus formation, as demonstrated in our pervious computational studies of thrombosis in TBAD (Menichini and Xu 2016; Menichini et al. 2016, 2018)). Within the PET for both P1 and P2, the mean and maximum TAWSS vary by up to 6% compared to the 3D IVP results, with the largest difference occurring when the flat profile is used. Other areas where differences are observed are near additional tears around the main abdominal branches in P1 and P2P, with areas of TAWSS differing usually by < 1 Pa. The results obtained with the non-patient-specific Flat75% and FlatP1 IVPs in P2 (Fig. 6b) show substantially lower TAWSS in many regions. Compared to the 3D IVP results, differences in maximum and mean TAWSS at the PET range from  − 27 to  − 35%. Within the descending aorta, the mean and maximum TAWSS values deviate by up to 27% when compared to the 3D IVP results.

### Pressure

**Fig. 8:**
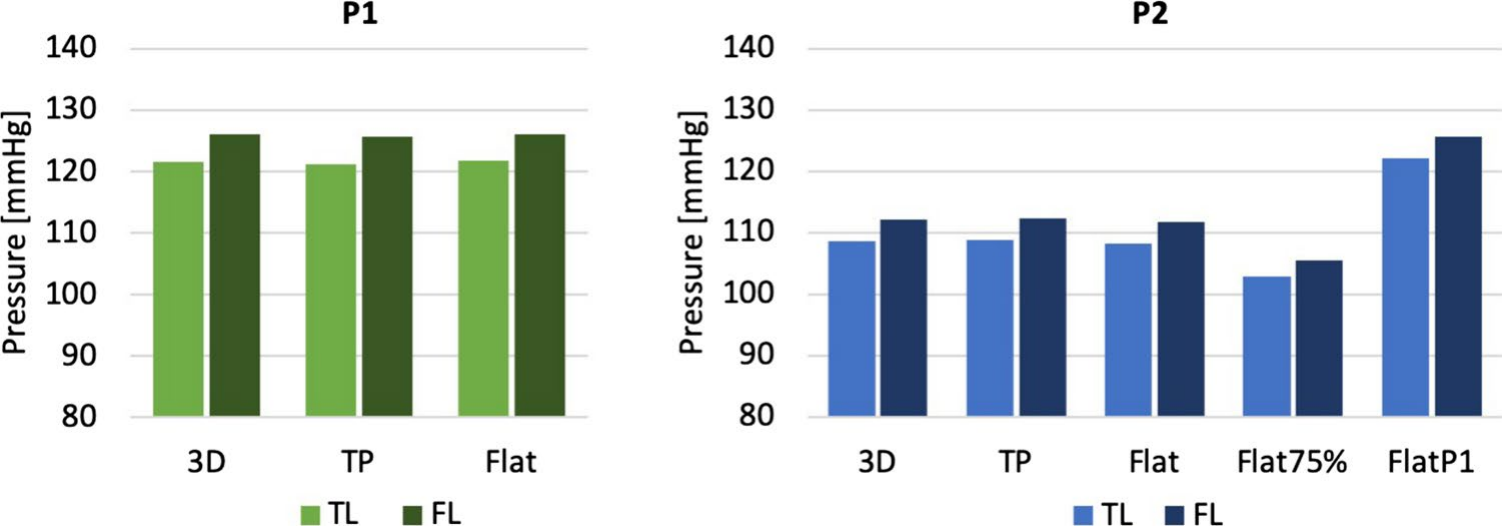
Average pressure within the true (TL) and false lumen (FL) at peak systole for P1, P2 and P2P, for all inlet velocity profiles applied

Figure 8 shows the spatially averaged pressure within the TL and FL at peak systole for P1 and P2 with all IVPs, while Table 1 gives the pressure difference between the FL and TL for each case—pressures were evaluated on planes shown in Fig. 1 and averaged within each lumen. It can be seen that there is little difference in the predicted TL or FL pressure between the patient-specific 3D, TP and flat IVPs, with a maximum difference of 1% across all models. In terms of pressure difference between the true and false lumen, using a TP IVP produced errors of up to 0.5% compared to the 3D IVP, while using a flat IVP produced errors of up to 6%. In P2, the results obtained with the Flat75% and FlatP1 IVPs are markedly different from those with the 3D IVPs, with errors of up to 6% and 13%, respectively, for luminal pressures, and errors of up to 25% and 6%, respectively, for pressure difference between the true and false lumen.

**Table 1:** Cross-lumen pressure difference (Δ*P*) for each patient with simulated inlet profiles

	$$\Delta P(P_{{{\text{FL}}}} - P_{{{\text{TL}}}} )\;[{\text{mmHg}}]$$				
	3D	TP	Flat	Flat75%	FlatP1
P1	4.6	4.5	4.3	–	–
P2	3.4	3.5	3.6	2.6	3.6
P2P	− 35.7	− 35.6	− 35.4	–	–

*FL* false lumen, *TL* true lumen

## Discussion

When a patient is diagnosed with type B aortic dissection, there are various treatment options which a clinician must navigate to achieve the optimal outcome. Patient-specific computational fluid dynamic analysis can potentially assist clinicians in their decision-making process, by providing in depth information on the hemodynamics within the aorta and predicting the potential outcome of various treatments. It can also help identify patients in need for urgent intervention or re-intervention after the initial treatment—for example, in cases where high FL pressures may lead to rapid FL expansion and potential aortic rupture.

Considerable efforts have been made to improve the clinical relevance and potential utility of CFD simulations. Developments in technology and computational methods have made it possible for 3D patient-specific inlet velocity profiles to be extracted from 4D MRI and applied as an inlet boundary condition. 3D velocity profiles contain velocity components in all three directions; hence, they are more detailed than a TP or flat profile but are not commonly available. Studies have shown that hemodynamics in the ascending aorta and aortic arch differ greatly between the results obtained with 3D, TP and flat IVPs (Morbiducci et al. 2013; Pirola et al. 2018; Youssefi et al. 2018). They also suggest that within the descending aorta the flow is developed and any differences due to the inlet profile are likely to have dissipated, resulting in similar predictions regardless of the shape of IVP. These studies, however, have been conducted in either healthy or aneurysmal aortas. The influence of inlet condition on type B dissection simulations specifically has not been reported prior to this study.

For most dissection patients, only CT scans acquired for diagnosis purposes are available, which do not contain any information on flow. In these cases, adopting a generic inlet flow or velocity waveform has been a common practice (Alimohammadi et al. 2013; Chen et al. 2013; Cheng et al. 2010; Dillon-Murphy et al. 2015; Tse et al. 2010). Therefore, the impact of applying a non-patient-specific inlet profile was also investigated in this study. This was done on P2 through modifying its flow waveform to simulate a 25% reduction in stroke volume, and also by applying the flow waveform for P1, both implemented through flat IVPs. These two additional simulations allowed for the effect of reduced stroke volume and a varied flow waveform to be analysed separately.

**Fig. 9:**
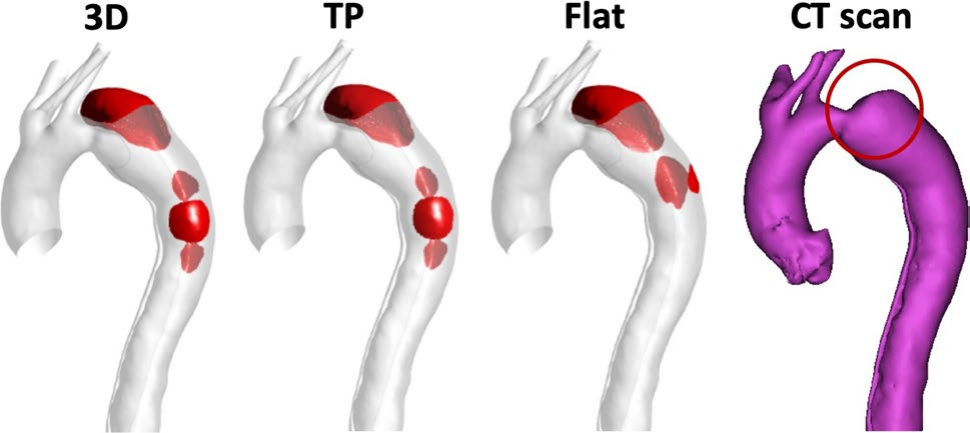
Predicted thrombus formation (shown in red) in P2 with a 3D, TP and Flat inlet velocity profile, alongside partially thrombosed (highlighted in red circle) geometry of P2 segmented from CT scan

Across all hemodynamic parameters (velocity, flow patterns and TAWSS), significant differences were observed in the ascending aorta of all geometric models when comparing the results obtained with different IVPs, reiterating previous findings that 3D IVPs are indispensable to faithful reproduction of flow characteristics in the ascending aorta (Chandra et al. 2013; Morbiducci et al. 2013; Pirola et al. 2018; Youssefi et al. 2018). Our results also showed that there were differences induced by the varied IVPs in the descending aorta, and these were confined to regions near the entry and re-entry tears. Closer inspection of the region around the PET in P1 and P2 revealed that while there was little notable difference in flow patterns, the absolute difference TAWSS contours (Figs. 5b and 6b) revealed discrepancies in the proximal FL around the PET. Values of TAWSS and instantaneous wall shear stress are crucial to the prediction of thrombus formation (Menichini and Xu 2016; Menichini et al. 2016, 2018), atherosclerosis (Alimohammadi et al. 2017) and retrograde dissection (Osswald et al. 2017). Therefore, it is necessary to determine to what extent such variations might affect the predicted thrombus formation. To this end, additional simulations were performed on P2 with our validated thrombosis model (Menichini and Xu 2016; Menichini et al. 2016, 2018). The results are shown in Fig. 9, and it can be seen that the main area of thrombosis in the proximal FL, identified in the follow-up CT scan also shown in Fig. 9, was well captured by all IVPs. The model also predicted additional thrombus formation in the thoracic FL, which is not evident in the CT scan. This may be attributed to possible differences between the reconstructed dissection geometry and its true original state, as reconstructing the pre-thrombus FL by simply removing the thrombus could have missed any changes in tear size and FL dimension.

Other idealised IVPs have been commonly used, such as parabolic and Womersley velocity profiles. Their influences on flow patterns and hemodynamic parameter have been studied in the aorta (non-TBAD) by various researchers (e.g. Youssefi et al. 2018; Morbiducci et al. 2013; Chandra et al. 2013). To avoid duplication of effort, parabolic and Womersley profiles were not included in this study. Nevertheless, the results obtained with these IVPs would be expected to be closer to those with the TP IVP than Flat IVP when the same flow waveform is used.

Simulations with the Flat75% and FlatP1 IVPs demonstrated the effect of using a non-patient-specific flow condition. The peak systolic flow rates for the 3D, FlatP1 and Flat75% IVP were 24.5, 22.6 and 18.4 L/min, respectively, and the peak velocity through the PET reflected these differences, with a smaller error being induced by the FlatP1 IVP than the Flat75% IVP. As the magnitude of wall shear stress is directly influenced by the flow rate, it is not surprising that TAWSS values are sensitive to the choice of flow waveform, especially the corresponding stroke volume. Using the two non-patient-specific flow waveforms caused errors of up to  − 35% in TAWSS in the PET and lower TAWSS throughout the descending aorta—in particular, there were larger areas below 0.2 Pa in the FL. Based on the threshold values in our thrombus prediction model, it is likely that thrombus would form throughout the FL in places it would not with the other IVPs. Therefore, using a non-patient-specific stroke volume would likely either over-predict or under-predict thrombus formation.

Comparisons with in vivo MRI flow data showed that all patient-specific IVPs (3D, TP and Flat) were able to reproduce flow through the PET both qualitatively and quantitatively. Closer examinations revealed that while all IVPs were adequate for reproducing the general flow pattern and shape of the high velocity jet through the PET, a smaller volume of high velocities was obtained with the flat IVP. Quantitative comparisons of peak systolic velocities through the PET demonstrated high level of agreement  − 0.9 m/s with all IVPs for P1, compared to 1.1 m/s from 4D flow MRI (Pirola et al. 2019); and 0.6 m/s with all IVPs for P2, compared to 0.7 m/s from 4D flow MRI. Finally, it is worth noting that the thoracic FL is characterised by slow flow, making it difficult to conduct quantitative comparisons due to large uncertainties in the 4D MRI data.

Pirola et al. (2019) also reported invasive Doppler wire pressure measurements for P1, which showed the TL to have a higher average pressure compared to the FL, with the difference being 2.3 mmHg. This is contradictory to the simulations in this study which predicted a higher pressure in the FL, with an average cross-lumen pressure of 4.6 mmHg for the 3D IVP. This discrepancy was also found by Pirola et al. (2019) in their CFD simulation of P1 and is likely attributed to the rigid-wall assumption which ignored the effect of flap motion. Considering the cross-lumen pressure difference predicted by the other patient-specific IVPs, for both P1 and P2, the TP IVP induced a negligible error, while the Flat IVP produced errors of up to 6%. In P2, both non-patient-specific IVPs predicted a higher-pressure FL with errors up to 25% using Flat75%, suggesting that the peak flow rate has a stronger influence on the predicted luminal pressure difference than the shape of flow waveform. Regarding the average pressure values within each lumen, comparisons for P2 (Fig. 8) clearly demonstrated the importance of the shape of flow waveform, in addition to stroke volume. The implication of these findings is that patient-specific flow waveforms should be used for reliable predictions of pressure and luminal pressure difference in TBAD.

The present study involves several limitations. First and foremost, the aortic wall and intimal flap were assumed to be rigid. The aorta is a compliant vessel and in the acute phase of dissection the intimal flap is known to be highly mobile (Peterss et al. 2016). This is particularly important for the models of P1 and P2 which simulated the early pre-TEVAR stage of the disease. Fluid–structure interaction (FSI) studies by Alimohammadi et al. (2015), Bäumler et al. (2020) and Qiao et al. (2019b) suggested that while FL flow was not qualitatively affected by the rigid wall assumption, substantial differences were noted in regions of low TAWSS between the rigid and FSI models. Furthermore, the dynamic mobility of intimal flap could have a strong influence on the predicted pressure values (Bäumler et al. 2020). The mechanical behaviour of stent-graft in post-TEVAR models has also been studied recently (Qiao et al. 2019a, 2020), which can be incorporated into the post-TEVAR model (P2P) in the future. Additionally, blood was assumed to be a Newtonian fluid in the CFD simulations presented here. While blood is known to exhibit non-Newtonian behaviour, its quantitative effect on flow patterns and hemodynamic parameters in TBAD has been investigated (Cheng et al. 2010), and the consistency across all simulations in this study negates any influence of viscosity when comparing IVPs.

## Conclusion

This study demonstrates the importance of the choice of inlet velocity profile in type B aortic dissection simulations. The results show that, qualitatively, there was little difference in TAWSS, velocity and flow patterns throughout the aorta when comparing patient-specific 3D, TP and flat IVPs. However, TAWSS values especially in the range between 0 and 1 Pa differed, with the flat IVP showing larger deviations from the results obtained with 3D IVPs. It was found that all essential hemodynamic parameters in type B aortic dissections could be predicted with good accuracy using TP IVPs. Hence, when patient-specific velocity data is available, a TP IVP should be used instead of a flat IVP. Using non-patient-specific flow waveforms produced significantly different results. The maximum velocity through the PET was strongly dependant on the peak systolic flow rate, while the simulated stroke volume had a direct influence on TAWSS. Predicted TL and FL pressures and luminal pressure difference were highly sensitive to the chosen peak systolic flow rate and the shape of flow waveform. Therefore, CFD results obtained with a generic flow waveform must be treated with caution when quantitative values of TAWSS and pressure are of interest. In the absence of 4D-flow MRI data, efforts should be made to obtain patient-specific stroke volume and adjust a generic flow waveform accordingly, even for qualitative analysis of hemodynamics in aortic dissections.

## Electronic supplementary material

Below is the link to the electronic supplementary material.

Supplementary file1 (PDF 350 kb)
