# Supplementary material for: The influence of inlet velocity profile on predicted flow in type B aortic dissection
Source: Biomech Model Mechanobiol. 2020 Oct 17;20(2):481–90. doi: 10.1007/s10237-020-01395-4 (PMC7979630; doi:10.1007/s10237-020-01395-4)
Supplement: Supplementary file 1 — Supplementary file1 (PDF 350 kb) [file 10237_2020_1395_MOESM1_ESM.pdf]

The influence of inlet velocity profile on predicted flow in type B aortic dissection

Armour et al.

SUPPLEMENTARY MATERIAL

Details on the mesh sensitivity tests conducted in this study can be found below. Qualitative visual comparison of time-averaged wall shear stress (TAWSS) contours was first conducted to identify any significant regions of inconsistent results. Quantitative comparison of mean and maximum velocity and (TAWSS) on selected planes, which are shown for each model in Figure 1, was then carried out and the results are reported below. The chosen mesh for each model had a % difference of < 3.5% for each hemodynamic parameter between the selected mesh and a more refined mesh, as well as a grid convergence index (GCI) of < 5.5%, in line with previous studies (Craven et al., 2009, Tedaldi et al., 2018) . GCI was calculated through the following equations (Craven et al., 2009).

$$r \approx \left(\frac{N_3}{N_2}\right)^{1/3} \approx \left(\frac{N_2}{N_1}\right)^{1/3}$$

$$p = \frac{\ln\left(\frac{|f_1 - f_2|}{|f_2 - f_3|}\right)}{\ln(r)}$$

$$E_3 = \frac{\left(\frac{|f_2 - f_3|}{f_3}\right)}{r^p - 1}, \qquad E_2 = \frac{\left(\frac{|f_1 - f_2|}{f_2}\right)}{r^p - 1}$$

$$GCI_{3,2} = F_S|E_3|, \qquad GCI_{2,1} = F_S|E_2|$$

Where $N_{1,2,3}$ is the number of elements in mesh M1 (coarse), M2 (medium) and M3 (fine), $f_{1,2,3}$ is the variable of interest (mean/max velocity or TAWSS), and F_S is the "factor of safety" equal to 1.25 (Craven et al., 2009). All chosen meshes are of a size consistent with previous studies (Auricchio et al., 2014, Cheng et al., 2010, Menichini et al., 2016, Menichini et al., 2018, Pirola et al., 2019, Romarowski et al., 2018, Wan Ab Naim et al., 2016).

Table 1: Number of elements in each mesh, coarse (M1), medium (M2) and fine (M3), for P1, P2 and P2P.

	**P1**	**P2**	**P2P**
**M1**	3,976,559	3,084,907	1,988,598
**M2**	6,168,045	5,755,630	4,055,893
**M3**	9,654,667	10,094,465	6,358,124

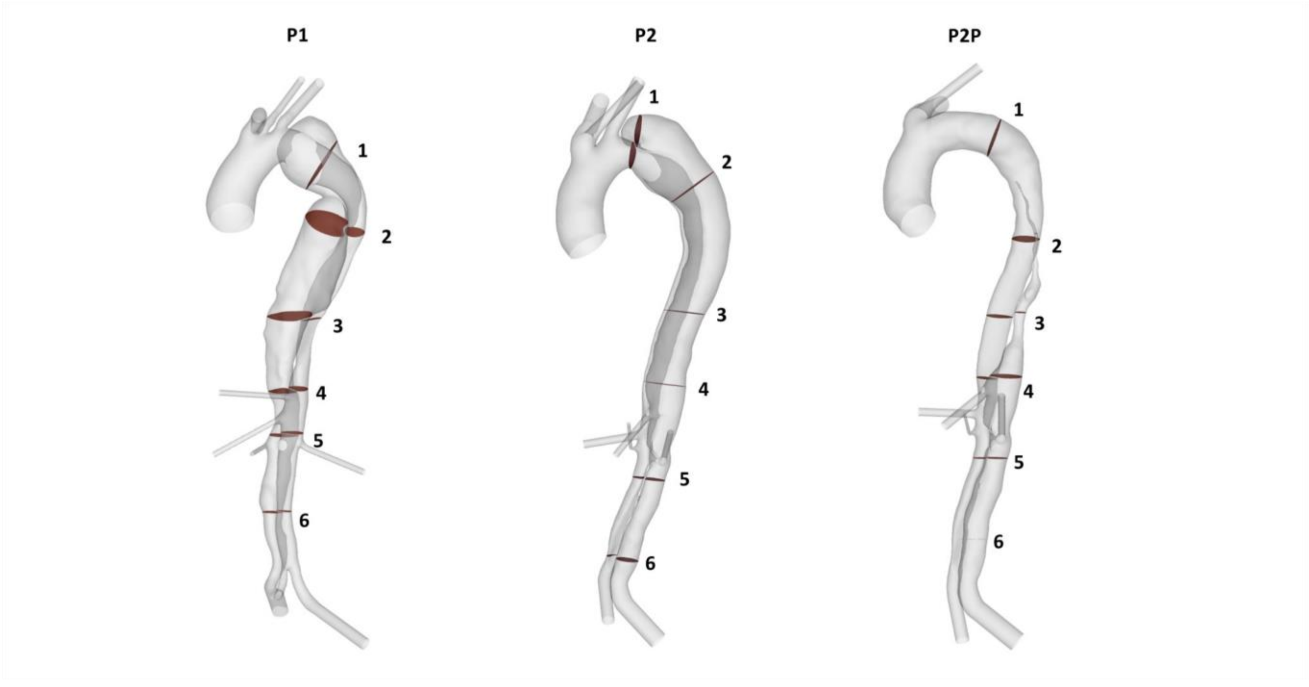

Figure 1: Analysis planes used for mesh sensitivity tests for models P1, P2 and P2P.

P1 mesh sensitivity tests

Table 2: Mean and maximum velocity on analysis planes (shown in Figure 1) in each mesh for P1. |%E|: Absolute percentage change in value between noted meshes. GCI: grid convergence index between noted meshes.

	Mean Velocity							Maximum Velocity					
**Plane**	1	2	3	4	5	6		1	2	3	4	5	6
**M1 [m/s]**	0.090	0.100	0.166	0.240	0.268	0.276		0.205	0.214	0.671	0.357	0.488	0.447
**M2 [m/s]**	0.090	0.100	0.166	0.241	0.277	0.274		0.204	0.212	0.668	0.358	0.490	0.445
**M3 [m/s]**	0.090	0.100	0.166	0.241	0.278	0.274		0.204	0.212	0.667	0.358	0.491	0.445
**|%E| M2/M1**	0.045	0.111	0.124	0.170	3.411	0.669		0.454	0.525	0.477	0.326	0.521	0.249
**|%E| M3/M2**	0.080	0.180	0.115	0.077	0.259	0.103		0.359	0.068	0.144	0.089	0.097	0.065
**$GCI_{2,1}$**	0.125	0.363	1.843	0.177	0.352	0.152		2.113	0.098	0.257	0.153	0.149	0.110
**$GCI_{3,2}$**	0.225	0.589	1.702	0.081	0.028	0.023		1.670	0.013	0.078	0.042	0.028	0.029

Table 3: Mean and maximum time-averaged wall shear stress (TAWSS) on analysis planes (shown in Figure 1) in each mesh for P1. |%E|: Absolute percentage change in value between noted meshes. GCI: grid convergence index between noted meshes.

	Mean TAWSS							Maximum TAWSS					
**Plane**	1	2	3	4	5	6		1	2	3	4	5	6
**M1 [Pa]**	0.740	0.219	1.088	0.578	0.726	0.786		1.536	0.530	3.961	1.208	1.807	1.709
**M2 [Pa]**	0.777	0.220	1.081	0.582	0.725	0.790		1.568	0.536	3.945	1.198	1.792	1.727
**M3 [Pa]**	0.793	0.219	1.085	0.570	0.738	0.799		1.589	0.537	3.958	1.160	1.797	1.737
**|%E| M2/M1**	4.953	0.081	0.624	0.568	0.080	0.515		2.084	1.233	0.405	0.804	0.826	1.033
**|%E| M3/M2**	2.110	0.100	0.422	2.031	1.800	1.191		1.339	0.153	0.341	3.150	0.307	0.577
**$GCI_{2,1}$**	4.772	0.531	1.606	0.978	0.104	1.124		4.861	0.219	2.647	1.364	0.607	1.657
**$GCI_{3,2}$**	2.090	0.657	1.074	3.590	2.313	2.582		3.145	0.027	2.212	5.474	0.223	0.930

Based on the mesh sensitivity results, M2 was chosen for P1.

P2 mesh sensitivity tests

Table 4: Mean and maximum velocity on analysis planes (shown in Figure 1) in each mesh for P2. |%E|: Absolute percentage change in value between noted meshes. GCI: grid convergence index between noted meshes.

	Mean Velocity							Maximum Velocity					
**Plane**	1	2	3	4	5	6		1	2	3	4	5	6
**M1 [m/s]**	0.089	0.071	0.131	0.121	0.139	0.190		0.318	0.146	0.168	0.185	0.190	0.465
**M2 [m/s]**	0.089	0.071	0.131	0.121	0.138	0.189		0.315	0.145	0.167	0.183	0.189	0.464
**M3 [m/s]**	0.089	0.071	0.131	0.121	0.138	0.189		0.316	0.145	0.168	0.182	0.189	0.460
**|%E| M2/M1**	0.266	0.018	0.063	0.051	0.765	0.753		0.693	0.472	0.770	1.023	0.688	0.140
**|%E| M3/M2**	0.101	0.012	0.072	0.121	0.173	0.191		0.043	0.059	0.415	0.118	0.115	0.785
**$GCI_{2,1}$**	0.077	0.027	0.715	0.262	0.063	0.080		0.003	0.010	0.596	0.019	0.029	1.204
**$GCI_{3,2}$**	0.203	0.042	0.626	0.111	0.280	0.319		0.057	0.084	1.118	0.167	0.173	0.213

Table 5: Mean and maximum time-averaged wall shear stress (TAWSS) on analysis planes (shown in Figure 1) in each mesh for P2. |%E|: Absolute percentage change in value between noted meshes. GCI: grid convergence index between noted meshes.

	Mean TAWSS						Maximum TAWSS					
**Plane**	1	2	3	4	5	6	1	2	3	4	5	6
**M1 [Pa]**	0.376	0.149	0.222	0.198	0.323	0.686	1.152	0.287	0.302	0.330	0.493	2.015
**M2 [Pa]**	0.389	0.149	0.222	0.198	0.323	0.687	1.191	0.291	0.304	0.328	0.481	2.012
**M3 [Pa]**	0.392	0.149	0.223	0.199	0.324	0.686	1.203	0.293	0.304	0.334	0.477	1.990
**|%E| M2/M1**	3.616	0.166	0.234	0.043	0.007	0.162	3.344	1.308	0.508	0.767	2.368	0.111
**|%E| M3/M2**	0.615	0.217	0.644	0.757	0.262	0.115	1.015	0.687	0.025	2.099	0.859	1.116
**GCI$_{2,1}$**	0.163	1.160	1.259	0.996	0.336	0.358	0.575	0.969	0.002	4.066	0.594	1.567
**GCI$_{3,2}$**	0.932	0.891	0.462	0.057	0.009	0.502	1.850	1.835	0.032	1.528	1.662	0.155

Based on the mesh sensitivity results, M2 was chosen for P2.

<u>P2P mesh sensitivity tests</u>

Table 6: Mean and maximum velocity on analysis planes (shown in Figure 1) in each mesh for P2P. |%E|: Absolute percentage change in value between noted meshes. GCI: grid convergence index between noted meshes.

	Mean Velocity						Maximum Velocity					
**Plane**	1	2	3	4	5	6	1	2	3	4	5	6
**M1 [m/s]**	0.097	0.222	0.285	0.127	0.091	0.119	0.147	0.267	0.397	0.507	0.249	0.364
**M2 [m/s]**	0.097	0.223	0.286	0.127	0.091	0.119	0.148	0.268	0.399	0.506	0.244	0.356
**M3 [m/s]**	0.097	0.223	0.285	0.127	0.091	0.119	0.148	0.268	0.400	0.506	0.244	0.354
**|%E| M2/M1**	0.337	0.302	0.371	0.636	0.114	0.074	0.225	0.562	0.462	0.104	2.002	2.262
**|%E| M3/M2**	0.064	0.063	0.116	0.055	0.056	0.025	0.125	0.124	0.285	0.072	0.020	0.560
**GCI$_{2,1}$**	0.098	0.100	0.212	0.075	0.140	0.048	0.349	0.200	0.940	0.291	0.025	0.924
**GCI$_{3,2}$**	0.019	0.021	0.067	0.007	0.069	0.016	0.194	0.044	0.582	0.201	0.000	0.225

Table 7: Mean and maximum time-averaged wall shear stress (TAWSS) on analysis planes (shown in Figure 1) in each mesh for P2P. |%E|: Absolute percentage change in value between noted meshes. GCI: grid convergence index between noted meshes.

	Mean TAWSS						Maximum TAWSS					
**Plane**	1	2	3	4	5	6	1	2	3	4	5	6
**M1 [Pa]**	0.153	0.331	0.477	0.381	0.383	0.735	0.252	0.513	0.804	1.335	1.165	2.204
**M2 [Pa]**	0.153	0.332	0.478	0.384	0.381	0.721	0.254	0.521	0.809	1.387	1.173	2.165
**M3 [Pa]**	0.153	0.333	0.477	0.387	0.381	0.718	0.256	0.521	0.806	1.393	1.187	2.166
**|%E| M2/M1**	0.041	0.371	0.227	0.660	0.291	1.880	0.946	1.490	0.659	3.863	0.669	1.780
**|%E| M3/M2**	0.341	0.288	0.262	0.854	0.025	0.460	0.612	0.111	0.342	0.470	1.191	0.045
**GCI$_{2,1}$**	0.058	1.623	2.122	3.532	0.035	0.756	2.210	0.149	0.893	0.673	1.877	0.057
**GCI$_{3,2}$**	0.483	1.260	2.456	4.560	0.003	0.182	1.436	0.011	0.468	0.085	3.327	0.001

Based on the mesh sensitivity results, M2 was chosen for P2P.

<u>References</u>

Auricchio F, Conti M, Lefieux A, Morganti S, Reali A, Sardanelli F, Secchi F, Trimarchi S, Veneziani A, Takizawa K, Bazilevs Y, Tezduyar TE (2014) Patient-specific analysis of post-operative aortic hemodynamics: A focus on thoracic endovascular repair (TEVAR). Comput Mech 54(4):943–953

Craven BA, Paterson EG, Settles GS, Lawson MJ (2009) Development and verification of a high-fidelity computational fluid dynamics model of canine nasal airflow. J Biomech Eng 131(9):091002

Cheng Z, Tan FPP, Riga CV, Bicknell CD, Hamady MS, Gibbs RGJ, Wood NB, Xu XY (2010) Analysis of Flow Patterns in a Patient-Specific Aortic Dissection Model. J Biomech Eng 132:051007

Menichini C, Cheng Z, Gibbs RGJ, Xu XY (2016) Predicting false lumen thrombosis in patient-specific models of aortic dissection. J Royal Soc Interface 13(124):1–11

Menichini C, Cheng Z, Gibbs RGJ, Xu XY (2018) A computational model for false lumen thrombosis in type B aortic dissection following thoracic endovascular repair. J Biomech 66:36–43

Pirola S, Guo B, Menichini C, Saitta S, Fu W, Dong Z, Xu XY (2019) 4D Flow MRI-Based Computational Analysis of Blood Flow in Patient-Specific Aortic Dissection. IEEE 66(12):3411-3419

Romarowski RM, Lefieux A, Morganti S, Veneziani A, Auricchio F (2018) Patient-specific CFD modelling in the thoracic aorta with PC-MRI–based boundary conditions: A least-square three-element Windkessel approach. Int J Numer Methods Biol Eng 34(11):e3134

Tedaldi E, Montanari C, Aycock KI, Sturla F, Redaelli A, Manning KB (2018) An experimental and computational study of the inferior vena cava hemodynamics under respiratory-induced collapse of the infrarenal IVC. Med Eng Phys 54:44-55

Wan Ab Naim WN, Ganesan PB, Sun Z, Liew YM, Qian Y, Lee CJ, Jansen S, Hashim SA, Lim E (2016) Prediction of thrombus formation using vortical structures presentation in Stanford type B aortic dissection: A preliminary study using CFD approach. App Math Mod 40(4):3115-3127
